# Evaluation of the effects of pandemic-related fears on anxiety and depression: the mediating roles of traumatic stress and loneliness

**DOI:** 10.1186/s40359-024-01880-w

**Published:** 2024-07-12

**Authors:** Pakize Gamze Erten Bucaktepe, Fethiye Akgül, Sercan Bulut Çelİk

**Affiliations:** 1 Department of Family Medicine, Dicle University Medical School, Diyarbakır, Turkey; 2 Department of Infectious Diseases and Clinical Microbiology, Batman Training and Research Hospital, Batman, Turkey; 3 Batman GAP Family Health Center, Provincial Health Directorate, Batman, Turkey

**Keywords:** Healthcare workers, COVID-19 related fears, Traumatic stress, Loneliness, Anxiety, Depression

## Abstract

**Background:**

The detrimental mental health effects which emerged from COVID-19 have profoundly affected healthcare workers (HCWs) worldwide. The aim of this study was to investigate the mediating effects of traumatic stress and loneliness on the fears of contracting and dying from COVID-19, and anxiety and depression of HCWs during the pandemic.

**Methods:**

A cross-sectional online survey was completed by HCWs in a province of Turkey. The Hospital Anxiety Depression Scale, Impact of Events Scale-Revised and numerical rating scales (for fears of COVID-19 and loneliness) were used and a bootstrap approach was used in the analyses with SPSS PROCESS macro software.

**Results:**

Of the HCWs evaluated, 150 (34.4%) were doctors, with a mean duration of work experience of 10.6 ± 7.5 years. The results indicated that fear of contracting COVID-19 was directly related to anxiety (β = 0.244, *p* < 0.001) and depression (β = 0.135, *p* < 0.01) and that traumatic stress and loneliness mediated the relationships between the fear of contracting COVID-19 and anxiety (β = 0.435, *p* < 0.001; β = 0.235, *p* < 0.001, respectively) and depression (β = 0.365, *p* < 0.001; β = 0.294, *p* < 0.001, respectively). The fear of dying from COVID-19 was determined to be directly associated with anxiety (β = 0.190, *p* < 0.001) but not with depression (β = 0.066, *p* = 0.116), and traumatic stress and loneliness mediated the relationships between the fear of dying from COVID-19 and anxiety (β = 0.476, *p* < 0.001; β = 0.259, *p* < 0.001, respectively) and depression (β = 0.400, *p* < 0.001; β = 0.311, *p* < 0.001, respectively).

**Conclusions:**

The study results demonstrated the important roles of traumatic stress and loneliness in exacerbating the negative consequences of fears of COVID-19 on anxiety and depression, and provide insights for identifying HCWs at greater risk.

## Introduction

The Coronavirus disease 2019 (COVID-19) pandemic had destructive social and psychological effects on the whole of society [1, 2]. In addition to the direct effects on physical health, the mental health of individuals was negatively affected by social restrictions, the closure of schools and workplaces, loss of income, reduced economic activity, fear of becoming ill, and the priority given to COVID-19 control and treatment in the use of resources [3].

In previous disease outbreaks, it has been shown that in contrast to all the other effects the psychological effects can continue for long after the outbreak and can even be permanent, thereby showing the magnitude of the problem [4–6]. Healthcare workers (HCWs) who shouldered the burden of the COVID-19 pandemic to a great extent, having to be more active because of the increased workload, were more affected than other sectors, and became more prone to affective disorders [7, 8]. Previous studies have emphasized that stress, depression, and anxiety were the main psychological problems determined most often in HCWs during the pandemic and that resilience has proven to be the key factor for mental health and well-being of HCWs [9–12]. These psychological problems can trigger alcohol and drug dependency, diminish quality of life, increase the risk of suicide, and even cause functioning impairments [8, 13, 14]. In a meta-analysis conducted in the same period, Turkey was reported to be among the countries with higher-than-pooled prevalence in respect of mental health [15]. Similarly, in another study conducted in Turkey during the first wave of the pandemic, it was reported that HCWs experienced high levels of depression, anxiety, and distress symptoms at 77.6%, 60.2%, and 76.4%, respectively [16]. That HCWs were negatively affected psychologically also meant that the whole of society was affected as a result of reduced performance and loss of work force [9, 17].

To be able to foresee outcomes and take the necessary precautions it is important to have a detailed understanding of the feelings of HCWs who carried a significant proportion of the burden during the COVID-19 pandemic. Therefore, the aim of this study was to explore the impact of pandemic-related fears on anxiety and depressive symptoms of HCWs and to construct mediating models in order to investigate the mediating roles of traumatic stress and feeling lonely and distant. In clinical sciences, the use of new-generation approaches based on mediating and moderating processes, which are the basis of testable theories, rather than medical disease models focussed on specific treatment protocols, has provided a breakthrough forming a bridge between environments and cultures and thus allowing the opportunity for integration [7]. Investigation with this method of the effects of the COVID-19 pandemic on the mental health of HCWs will provide reliable evidence for the determination of needs and the support of mental health, and will allow early and effective interventions to be made in similar situations which may occur in the future.

### Fear, anxiety and depression

Fear, an unpleasant instinctive feeling that is felt when a person is threatened by an external or internal stimulus, is a short-lived present-oriented immediate response which is appropriate to an actual, clearly explained, specific threat [18, 19]. It is a type of defence mechanism related to an autonomic stimulus required for fight or flight, which is a basis for survival [2, 20]. Uncertain and ambiguous situations, such as a pandemic, may cause fear and worry about becoming infected and negative consequences of the infection. Nosophobia, the fear of becoming ill, became more important during the COVID-19 pandemic, and the probability of HCWs being affected was greater as they were at particular risk [19, 21]. The context-process-outcome model posits that the interpretation of events may vary depending on an individual’s cognitive processes [22]. It has been reported that emotions such as fear given in response to events and situations can form positive (psychological growth) or negative (psychopathologies) reactions depending on the interpretation in the consciousness of the interaction with personal experience, coping strategies, and defence mechanisms [23]. Therefore, although fear of the virus causes effects of different form and severity in everyone, it basically shows the same characteristics. If fear is chronic and excessive, as in the pandemic, the response of the individual to fear may not be functional, and this can cause anxiety [2, 24, 25]. According to the emotion dysregulation model, individuals who exhibit a negative hyperarousal state can experience problems in regular and appropriate management of mood expressions and control, and this can render them more vulnerable to anxiety disorders [26]. From the starting point of this model, it can be implied that those who experience greater fear of COVID-19 will be more predisposed to anxiety. The James Lange Theory of Emotions suggests that stimuli that cause visceral changes reaching as far as the cerebral cortex are perceived as emotion [23]. It was reported that there are three basic components of physiological, psychological, and behavioural of even basic emotions such as fear [23]. Thus, fears that create the perception of uncertainty and lack of control cause the feeling of anxiety, and by making coping more difficult this can create affective problems [23].

In this context, the first two hypotheses of this study were formed as follows:

#### H1

Fear of contracting COVID-19 is positively related to anxiety.

#### H2

Fear of dying from COVID-19 is positively related to anxiety.

In the learned helplessness theory, when an individual is in a situation beyond their control, they first feel helpless and then attribute this to internal, stable, and unchangeable factors such as their own capabilities [27]. By generalising the situation over time with evidence that they cannot do anything in any stressful or negative situation, the individual displays depressive symptoms. Accordingly, challenging life events such as pandemics and the feeling of helplessness created with widespread and intense fear may contribute to the development of depressive symptoms. In addition, due to the common negative effect, neurobiological processes, and shared similar diathesis, depression and anxiety resemble each other and can show a relationship with the same situations [28].

Therefore, it was hypothesised that:

#### H3

Fear of contracting COVID-19 is positively related to depression.

#### H4

Fear of dying from COVID-19 is positively related to depression.

### Mediating effect of traumatic stress

Lazarus and Folkman [29] defined stress as *“a particular relationship between the person and the environment that is appraised by the person as taxing or exceeding his or her resources and endangering his or her well-being”*. The generalised unsafety theory of stress (GUTS), interpreted from the neurobiological and evolution-theoretical perspective, suggests that stress is a “default response” which is below subcortical inhibition of the stress response and uncertainty of safety situations can cause disinhibition in this response, and it has been claimed that a real threat is not even necessary and that the perception of general unsafety is enough for activation [30]. Thus it was assumed that the safety signal of HCWs was violated by the knowledge of the presence of a dangerous virus from which they could not remove themselves because of their work requirements, and this caused stress.

The pandemic was a more significant source of stress for HCWs, and it has been reported that acute stress, distress, and post-traumatic stress symptoms increased [9, 31–33]. It has been reported that prefrontal cortex functions such as the organisation of planning, attention, and problem-solving skills are temporarily suspended during stress response [34]. Any kind of stress can disrupt the homeostasis that creates stability between all the biological and mental systems and stability of the regulatory systems [34]. The persistence of stress can lead to a series of biological and sociopsychological effects and may be an importaant antecedent of anxiety [34].

According to the transactional theory of stress and coping, the interpretation of stressful events such as pandemics is more important regarding the psychopathologies, than the events themselves [29]. Moreover, according to the context-process-outcome model, the outcomes created in the consciousness of the perceptions of the event causing stress can be more harmful than the effects of stress [22].

The transactional model states that as people constantly evaluate the stimuli around them, emotions are formed as a result of these evaluations [35]. If a stimulus is interpreted as threatening, challenging, or harmful, attempts are made to cope with it, but if this situation cannot be coped with, potentially harmful or damaging distress occurs [35]. Moreover, the theory of cognitive activation of stress posits that if a stimulus (the virus in the current study model) is perceived as threatening by the individual (self-appreciation), this causes an increase in brain activity by creating stress [36]. Thus it creates physiological, psychological, and behavioural effects and these effects can vary according to the experience, perception and interpretation of stress of each individual. Therefore, just as there may be no apparent effect, psychological effects such as anxiety, depression, burnout, and insomnia may develop [36]. Moreover, such serious events can cause depression very rapidly and it has been reported that a short period such as 3–4 weeks may be sufficient for the emergence of depressive symptoms [31, 37].

Therefore, the following hypotheses were formed:

#### H5

Traumatic stress mediates the relationship between fear of contracting COVID-19 and anxiety.

#### H6

Traumatic stress mediates the relationship between fear of contracting COVID-19 and depression.

#### H7

Traumatic stress mediates the relationship between fear of dying from COVID-19 and anxiety.

#### H8

Traumatic stress mediates the relationship between fear of dying from COVID-19 and depression.

### The mediating effects of feeling lonely and distant from others

Due to their working conditions, all HCWs encounter more infectious agents than the general population, and this increases the likelihood of both contracting and spreading disease. This can lead to stigmatisation by society and even by family [8, 38]. It is known that when stigmatized people are avoided by those in their social environment, reactive behaviours such as abstaining from social contact and self-isolation can be seen [39].

The World Health Organization (WHO) and International Labour Organization (ILO) have cited social isolation and stigmatisation as being mental health risk factors for HCWs during the COVID-19 pandemic, and emphasized that this rendered them especially more vulnerable to anxiety, depression, and insomnia [40].

Loneliness is the feeling of being socially isolated and distant from one’s environment as a result of the difference felt between the real social relationships that exist and the relationships that the person perceives and/or wants to have [41, 42]. Moreover, loneliness is known to be related to all-cause mortality and physical and mental health problems [42–45].

According to GUTS, compromised social networks such as loneliness can develop generalized uncertainty which is synonymous with unsafety, and intolerance of uncertainty may cause distress [30]. It may be difficult to differentiate loneliness from the physiological responses to stress that have formed in the individual, and GUTS attributes this to disinhibition of the default stress response [46].

According to the Evolutionary Theory of Loneliness (ETL), loneliness might have evolved as an aversive state just like hunger, thirst and pain, which serves to increase the likelihood of survival by promoting useful social connections [47, 48]. At the same time, this perception of social isolation can produce an implicit hypervigilance towards social threats, which leads to attentional, confirmatory and memory biases [49]. Cacioppo and Cacioppo [48] emphasized that although loneliness can encourage social behaviours for mutual benefit, it can also lead to problems in the modern world. According to this, individual differences can affect perceived social isolation, in other words the sensitivity to loneliness, and negative perception may cause very serious mental and physical symptoms [48].

The extreme loneliness felt because of fear and isolation during the pandemic may have formed maladaptive behavioural patterns causing greater hypervigilance and cognitive bias, and had a negative effect on mental health [50]. The mental health issues most associated with loneliness are said to be anxiety and depression [50]. According to the social allostatic load model, loneliness and the effects created form an allostatic burden, and if the individual cannot cope with this situation and if the event is prolonged, the allostatic burden can increase and depressed mood can develop due to allostatic overload [51].

Therefore, the following hypotheses were established:

#### H9

Feeling lonely and distant to others mediates the relationship between fear of contracting COVID-19 and anxiety.

#### H10

Feeling lonely and distant to others mediates the relationship between fear of contracting COVID-19 and depression.

#### H11

Feeling lonely and distant to others mediates the relationship between fear of dying from COVID-19 and anxiety.

#### H12

Feeling lonely and distant to others mediates the relationship between fear of dying from COVID-19 and depression.

## Materials and methods

### Participants and procedure

This descriptive, cross-sectional study was conducted on actively working HCWs (doctors, nurses, radiographers, laboratory technicians etc.) in hospitals in the province of Batman in southeast Turkey between July and September 2020. Approval for the study was granted by the Ethics Committee of Batman Regional State Hospital (2020/244). The study was conducted following the 2013 Helsinki Declaration ethical guidelines. The sample size was calculated using G*Power 3.1.9.7 software. The sample size required for correlation of the bivariate normal model was calculated to be at least 138 by a priori analysis. The calculation was made with a power of 0.95, correlation ρ H1 of 0.3, Correlation ρ H0 of 0 and α error probability of 0.05. Participation in the study was on a voluntary basis without the implementation of any inclusion and exclusion criteria. Taking into consideration the long hours worked by HCWs and the need to preserve social distancing, the questionnaire was delivered online via smartphones. The Google Forms program was used to create the questionnaire and the URL link was sent to HCWs using Whatsapp, the most preferred social networking application in Turkey. Information about the study, a sociodemographic data form including questions about age, gender, profession etc., numeric rating scales for fears related to COVID-19, a question about loneliness, and the scales (The Hospital Anxiety Depression Scale for anxiety and depression and Impact of Events Scale-Revised for stress) were uploaded to Google Forms, and an informed consent form consisting of purpose, procedures and requirements of the survey for participation in the study was also included. The participants were informed that their participation was voluntary, they could withdraw at any point and their responses would remain confidential and anonymous. The questionnaire was designed so that the participant had to first read the explanations about the study in the informed consent section and provide informed consent after fully understanding the procedure and before starting to complete the questionnaire. From a total of approximately 3000 HCWs working in two state hospitals and five private hospitals in the province of Batman, 517 were reached through the convenience sampling method. The response rate was approximately 17%. Of these, 81 were excluded from the study as the responses were determined to have come from the same IP or had been completed in less than 180 s. Finally, 436 questionnaires were included in the evaluations. Post-hoc power analysis indicated that the sample size used in this study had sufficient power for the proposed analysis. All methods were performed in accordance with the STROBE checklist to improve the quality of the article.

### Measures

#### Fear of contracting COVID-19 and fear of dying from COVID-19

To determine the fear of contracting COVID-19 and the fear of dying from COVID-19, two separate Numeric Rating Scales (NRS) were used, graded from 0 to 10. The NRS was reported to be valid, reliable and sensitive by Becker et al. [52]. Grading on the scale ranged from 0 = I am not afraid to 10 = I am very much afraid, with higher points indicating greater fear.

#### Anxiety and depression

The Hospital Anxiety Depression Scale (HADS) was used for this measurement. The HADS was developed by Zigmond and Snaith [53] to determine the risk of an individual in respect of anxiety and depression, and to measure the level and severity. The scale was adapted to Turkish by Aydemir [54]. The HADS consists of a total of 14 items with 4-point Likert-type responses, 7 of which (odd numbers) measure anxiety and 7 (even numbers) measure depression. A previous study in Turkey determined the cutoff points as 10/11 for the anxiety subscale and 7/8 for the depression subscale. Higher points indicate a higher risk. In this study, the internal consistency coefficient (ICC) (Cronbach α) was determined as 0.864 for the anxiety section and 0.820 for the depression section.

#### Traumatic stress

To measure the level of psychological stress in traumatic events encountered, the widely used Impact of Events Scale-Revised (IES-R), was applied to the HCWs to determine the stress created by COVID-19. The scale was developed by Weiss and Marmar [55] and adapted to Turkish by Çorapçıoğlu et al. [56]. In 3 subscales there are 22 items with Likert-type responses scored from 0 (never) to 4 (very much) to determine the severity of stress in the last 7 days. The cutoff points of the Turkish version of the scale are taken as 24–33 points, with higher points indicating greater stress. The evaluations were made on the total points of the scale. For this sample, the ICC (Cronbach α) was determined to be 0.934.

#### Feeling lonely and distant from others

To determine if the HCWs perceived loneliness, with prejudice and stigma towards them from society and the degree of this, the following question was asked: “During this period have you felt alone and distant from others?”. The responses were scored as 1 = No, 2 = Occasionally, 3 = Sometimes, 4 = Often, 5 = Always. The score was taken as a continuous variable in the evaluation with higher scores indicating greater loneliness.

### Data analysis

The data were extracted from Google Forms to Excel files, prepared for analysis, and uploaded to the SPSS program. Data obtained in the study were analyzed statistically using IBM SPSS vn. 27.0 software (IBM Corporation, Armonk, NY, USA), its plug-in PROCESS Macro vn. 4.0 program [57] and G*Power 3.1.9.7 software (Universität Düsseldorf: Psychologie-HHU). Descriptive tests were performed for demographic data. Conformity of the data to normal distribution was assessed with the skewness and kurtosis indicators. Continuous variables were stated as mean ± standard deviation values since the distributions of the variables were normal (the indices were between − 1 and + 1) and categorical variables were stated as number and percentage. There were no missing data because the items were set as required, so that the next section cannot be answered and the questionnaire cannot be completed if any item was left unmarked in the previous section. To determine relationships between measures and their 95% confidence interval (CI), Pearson correlation analysis was used. Multicollinearity was assessed using Tolerance and Variance Inflation Factor (VIF) tests and no problems were indcated as all values for VIF were less than 10 and for Tolerance test were greater than 0.2. The regression-based PROCESS model 4 was used to test the mediation effect and eight mediation analyses were performed with anxiety and depressive symptoms as dependent variables, COVID-19-related fears as independent variables, and loneliness and traumatic stress as mediators. In this method, the model was tested with the mediation significance bootstrapping procedure with 95% CI and random 5000 sampling. The 95% bias-corrected CI not containing zero in the results of the analysis means that the mediation effect is significant. To mitigate confounding bias, age and gender were added to the model as covariates. Harman’s single factor analysis method was applied to test common method bias [58]. Internal consistency was determined using the Cronbach α indicators, shown in the measures section. The results were assessed with a significance level (two tailed) of 0.05.

## Results

### Common method bias test

Harman’s analysis showed that the first factor explained 35.747% of the variance indicating no serious common method deviation, since the result was under the threshold value of 50%.

### Descriptive and correlation analysis

The total 436 participants comprised 230 (52.8%) males and 206 (47.2%) females, with a mean age of 34.6 ± 7.5 years (20–56 years). The mean years of experience were 10.6 ± 7.5 (range, 0.2–35 years), and 150 (34.4%) participants were doctors and 159 (36.5%) were nurses. The mean values and correlations with 95% CI of the scales used are shown in Table 1. The results showed that fear of contracting COVID-19, fear of dying from COVID-19, feeling lonely and distant from others, and traumatic stress were positively and significantly correlated to both anxiety and depression.

**Table 1:** Means and correlations of main variables

Variables	1	2	3	4		5	6
1- Anxiety (95% CI)							
2- Depression (95% CI)	0.732** (0.685–0.773)						
3- Traumatic stress (95% CI)	0.671** (0.616–0.720)	0.531** (0.460–0.595)					
4- Feeling lonely and distant (95% CI)	0.510** (0.437–0.576)	0.472** (0.395–0.542)	0.381** (0.298–0.459)				
5- Fear of contracting COVID-19 (95% CI)	0.561** (0.493–0.622)	0.410** (0.329–0.485)	0.494** (0.419–0.562)	0.356** (0.271–0.436)			
6- Fear of dying from COVID-19 (95% CI)	0.455** (0.378–0.527)	0.288** (0.200-0.372)	0.379** (0.295–0.456)	0.259** (0.169–0.344)		0.715** (0.666–0.758)	
Mean ± Standard Deviation	8.2 ± 4.6	8.4 ± 4.5	38.4 ± 17.7	2.9 ± 1.2		5.4 ± 3.1	4.8 ± 3.4

**p < 0.01, CI: confidence interval

### Tests of mediation effects

**Fig. 1:**
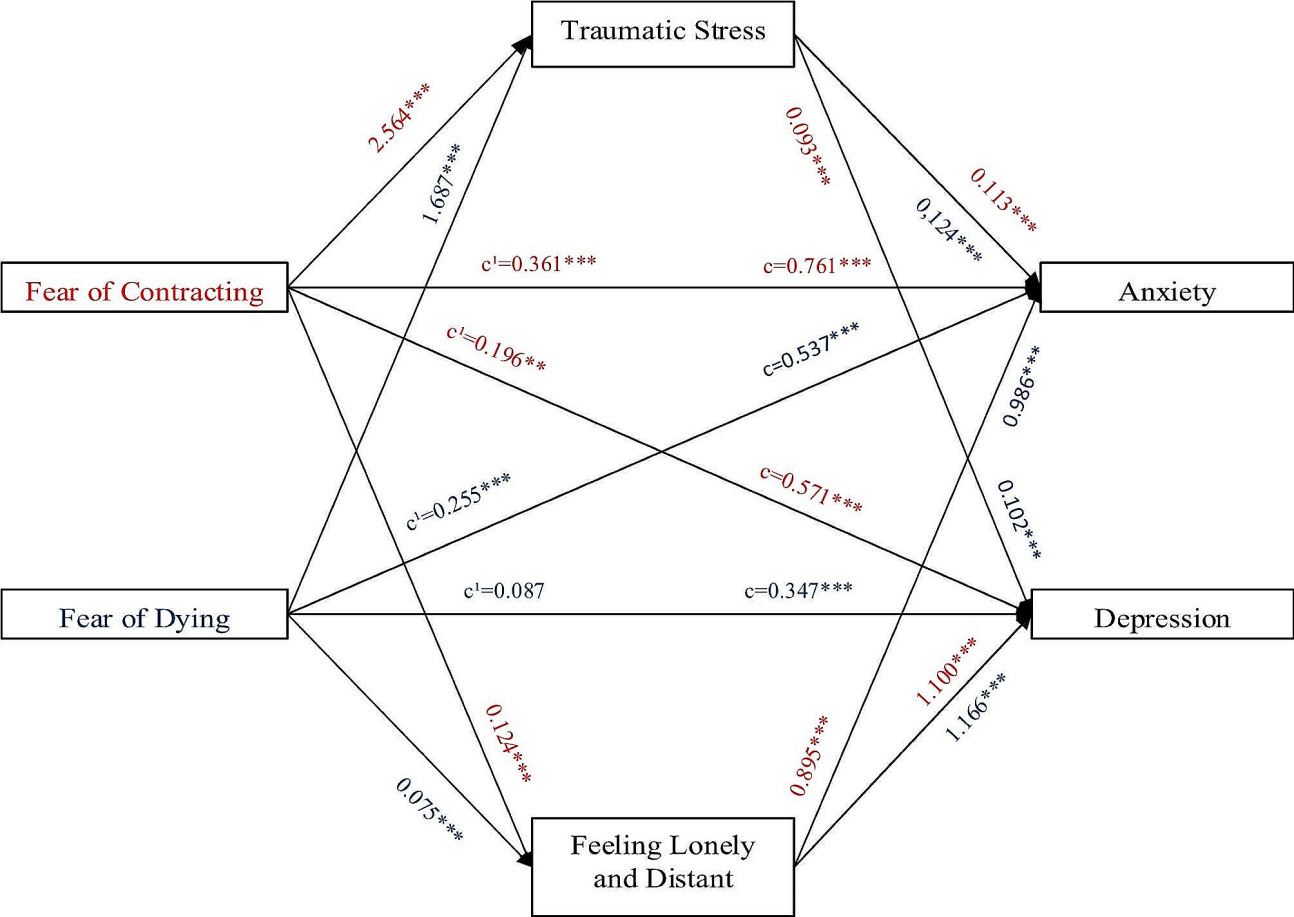
Parallel mediating model of traumatic stress and feeling lonely and distant in association between fears of COVID-19 and anxiety, and between fears of COVID-19 and depression. (Unstandardized coefficients are used. **p < 0.01, ***p < 0.001)

All the mediation models of the study are presented in Fig. 1, together with unstandardized path coefficients.

#### Testing the mediating effects of traumatic stress and feeling lonely and distant from others between the fear of contracting COVID-19 and anxiety

The results showed that fear of contracting COVID-19 was directly and positively related to anxiety (β = 0.244, *p* < 0.001), and positively related to traumatic stress and feeling lonely and distant from others (β = 0.449, *p* < 0.001; β = 0.318, *p* < 0.001, respectively). Both traumatic stress and feeling lonely and distant from others had significant relationships with anxiety (β = 0.435, *p* < 0.001; β = 0.235, *p* < 0.001, respectively) (Table 2). The statistical power of this model (0.581) was found to be above the large level of the standard R² value of 0.26 proposed by Cohen (Table 2).

**Table 2:** Mediating effects of traumatic stress and feeling lonely and distant from others between fear of contracting COVID-19 and anxiety, and between fear of contracting COVID-19 and depression

Regression equation		Goodness of fit		Significance				95% Confidence Interval	
Outcome	Predictor	R²	F (df1,df2)	B	SE	β	t	Lower	Upper
Traumatic stress	Fear of contracting	0.284	56.991 (3, 432)	2.564	0.239	0.449	10.744***	2.095	3.033
	Gender			-5.773	1.514	-0.163	-3.814***	-8.749	-2.798
	Age			-0.210	0.100	-0.089	-2.094*	-0.406	-0.013
Feeling lonely and distant	Fear of contracting	0.156	26.622 (3, 432)	0.124	0.018	0.318	7.014***	0.089	0.158
	Gender			-0.310	0.112	-0.129	-2.772**	-0.530	-0.090
	Age			-0.015	0.007	-0.090	-1.968	-0.029	0.000
Anxiety	Fear of contracting	0.581	119.107 (5, 430)	0.361	0.055	0.244	6.620***	0.254	0.468
	Traumatic stress			0.113	0.010	0.435	11.488***	0.093	0.132
	Feeling lonely and distant			0.895	0.133	0.235	6.741***	0.634	1.156
	Gender			-0.295	0.308	-0.032	-0.960	-0.900	0.309
	Age			-0.057	0.020	-0.093	-2.859**	-0.097	-0.018
Depression	Fear of contracting	0.385	53.738 (5, 430)	0.196	0.065	0.135	3.021**	0.069	0.324
	Traumatic stress			0.093	0.012	0.365	7.963***	0.070	0.116
	Feeling lonely and distant			1.100	0.158	0.294	6.954***	0.789	1.411
	Gender			0.672	0.366	0.074	1.883	-0.048	1.391
	Age			-0.026	0.024	-0.042	-1.068	-0.073	0.021

*p < 0.05, **p < 0.01, ***p < 0.001, B: unstandardized coefficients, SE: standard error, β: standardized coefficients

The bootstrap analysis results of the multiple mediating effects between fear of contracting COVID-19 and anxiety are presented in Table 3. The total, direct and total indirect effects were determined to be 0.513, 0.243 and 0.270 respectively and significant, since the upper and lower levels of 95% confidence intervals do not include zero. The total indirect effects accounted for 53% of the total effects, as 38% for traumatic stress and 15% for feeling lonely and distant.

#### Testing the mediating effects of traumatic stress and feeling lonely and distant from others between the fear of contracting COVID-19 and depression

The results showed that fear of contracting COVID-19 was directly and positively related to depression (β = 0.135, *p* < 0.01), and positively related to traumatic stress and feeling lonely and distant from others (β = 0.449, *p* < 0.001; β = 0.318, *p* < 0.001, respectively). Both traumatic stress and feeling lonely and distant from others had significant relationships with depression (β = 0.365, *p* < 0.001; β = 0.294, *p* < 0.001, respectively) (Table 2). The statistical power of this model (0.385) was above the large level of the standard R² value of 0.26 proposed by Cohen (Table 2).

The bootstrap method tested the significance of the multiple mediating effects between fear of contracting COVID-19 and depression, and the results are presented in Table 3. The total, direct and total indirect effects were 0.392, 0.135 and 0.257 respectively and significant, since the upper and lower levels of 95% confidence intervals do not include zero. The total indirect effects accounted for 66% of the total effects, as 42% for traumatic stress and 24% for feeling lonely and distant.

**Table 3:** Bootstrap analysis of multiple mediation effects between fear of contracting COVID-19 and anxiety, and between fear of contracting COVID-19 and depression

		Effect size (Standardized)	Standard Error	Percentage of total effects	95% Confıdence Interval	
					Lower	Upper
Between fear of contracting COVID-19 and anxiety	Total effects	0.513	0.058	100%	0.646	0.875
	Direct effects	0.243	0.055	47%	0.254	0.468
	Total Indirect effects	0.270	0.028	53%	0.217	0.328
	Traumatic stress	0.195	0.024	38%	0.149	0.245
	Feeling lonely and distant	0.075	0.015	15%	0.048	0.106
Between fear of contracting COVID-19 and Depression	Total effects	0.392	0.065	100%	0.443	0.699
	Direct effects	0.135	0.065	34%	0.069	0.324
	Total Indirect effects	0.257	0.030	66%	0.199	0.318
	Traumatic stress	0.164	0.025	42%	0.118	0.215
	Feeling lonely and distant	0.093	0.019	24%	0.060	0.133

#### Testing the mediating effects of traumatic stress and feeling lonely and distant from others between the fear of dying from COVID-19 and anxiety

The results showed that fear of dying from COVID-19 was directly and positively related to anxiety (β = 0.190, *p* < 0.001), and positively related to traumatic stress and feeling lonely and distant from others (β = 0.326, *p* < 0.001; β = 0.214, *p* < 0.001, respectively). Both traumatic stress and feeling lonely and distant from others had significant relationships with anxiety (β = 0.476, *p* < 0.001; β = 0.259, *p* < 0.001, respectively) (Table 4). The statistical power of this model (0.568) was above the large level of the standard R² value of 0.26 proposed by Cohen (Table 4).

**Table 4:** Mediating effects of traumatic stress and feeling lonely and distant between fear of dying from COVID-19 and anxiety, and between fear of dying from COVID-19 and depression

Regression equation		Goodness of fit		Significance				95% Confidence Interval	
Outcome	Predictor	R²	F (df1,df2)	B	SE	β	t	Lower	Upper
Traumatic stress	Fear of dying	0.193	34.455 (3, 432)	1.687	0.230	0.326	7.352***	1.236	2.138
	Gender			-6.530	1.607	-0.185	-4.062***	-9.689	-3.371
	Age			-0.229	0.106	-0.097	-2.152*	-0.438	-0.020
Feeling lonely and distant	Fear of dying	0.103	16.603 (3, 432)	0.075	0.017	0.214	4.576***	0.043	0.108
	Gender			-0.354	0.115	-0.147	-3.070**	-0.581	-0.127
	Age			-0.016	0.008	-0.098	-2.065*	-0.031	-0.001
Anxiety	Fear of dying	0.568	113.038 (5, 430)	0.255	0.047	0.190	5.460***	0.163	0.346
	Traumatic stress			0.124	0.010	0.476	12.943***	0.105	0.142
	Feeling lonely and distant			0.986	0.133	0.259	7.408***	0.724	1.247
	Gender			-0.246	0.313	-0.027	-0.786	-0.861	0.369
	Age			-0.054	0.020	-0.088	-2.663**	-0.094	-0.014
Depression	Fear of dying	0.375	51.626 (5, 430)	0.087	0.055	0.066	1.577	-0.021	0.195
	Traumatic stress			0.102	0.011	0.400	9.033***	0.080	0.124
	Feeling lonely and distant			1.166	0.157	0.311	7.415***	0.857	1.476
	Gender			0.667	0.370	0.074	1.803	-0.060	1.395
	Age			-0.025	0.024	-0.041	-0.033	-0.072	-0.230

*p < 0.05, **p < 0.01, ***p < 0.001, B: unstandardized coefficients, SE: standard error, β: standardized coefficients

The bootstrap analysis results of the multiple mediating effects between fear of dying from COVID-19 and anxiety are presented in Table 5. The total, direct and total indirect effects were 0.401, 0.190 and 0.211 respectively and significant, since the upper and lower levels of 95% confidence intervals do not include zero. The total indirect effects accounted for 53% of the total effects, as 39% for traumatic stress and 14% for feeling lonely and distant.

**Table 5:** Bootstrap analysis of multiple mediation effects between fear of dying from COVID-19 and anxiety, and between fear of dying from COVID-19 and depression

		Effect size (Standardized)	Standard Error	Percentage of total effects	95% Confıdence Interval	
					Lower	Upper
Between fear of dying from COVID-19 and anxiety	Total effects	0.401	0.057	100%	0.426	0.649
	Direct effects	0.190	0.047	47%	0.163	0.346
	Total Indirect effects	0.211	0.028	53%	0.207	0.365
	Traumatic stress	0.155	0.024	39%	0.146	0.278
	Feeling lonely and distant	0.055	0.014	14%	0.038	0.115
Between fear of dying from COVID-19 and depression	Total effects	0.263	0.062	100%	0.225	0.468
	Direct effects	0.066	0.055	25%	-0.021	0.195
	Total Indirect effects	0.197	0.030	75%	0.140	0.258
	Traumatic stress	0.131	0.024	50%	0.086	0.181
	Feeling lonely and distant	0.067	0.018	25%	0.034	0.102

#### Testing the mediating effects of traumatic stress and feeling lonely and distant from others between the fear of dying from COVID-19 and depression

The results showed that fear of dying from COVID-19 was not directly related to depression (β = 0.066, *p* = 0.116), and was positively related to traumatic stress and feeling lonely and distant from others (β = 0.326, *p* < 0.001; β = 0.214, *p* < 0.001, respectively). Both traumatic stress and feeling lonely and distant from others had significant relationships with depression (β = 0.400, *p* < 0.001; β = 0.311, *p* < 0.001, respectively) (Table 4). The statistical power of this model (0.375) was above the large level of the standard R² value of 0.26 proposed by Cohen (Table 4).

The bootstrap analysis results of the multiple mediating effects between fear of dying COVID-19 and depression are presented in Table 5. The total, direct and total indirect effects were 0.263, 0.066 and 0.197 respectively, and the total and total indirect effects were significant, since the upper and lower levels of 95% confidence intervals do not include zero. The total indirect effects accounted for 75% of the total effects, as 50% for traumatic stress and 25% for feeling lonely and distant.

## Discussion

Using an online questionnaire delivered to HCWs, the current study examined the relationships between COVID-19 related fears and anxiety, and depression during the first wave of the pandemic, and tested the mediating effects of traumatic stress and feeling lonely and distant on these associations. The results indicated that fear of contracting COVID-19 and fear of dying from COVID-19 had an impact on anxiety and depressive symptoms of HCWs. In addition, the analysis suggested that both traumatic stress and feeling lonely and distant from others had parallel mediating roles between either fear of contracting COVID-19 or fear of dying from COVID-19 and anxiety. The results also proved similar mediating roles for depression.

### The effects of fear of contracting COVID-19 and fear of dying from COVID-19 on anxiety and depression

The correlation analysis results showed that fear of contracting COVID-19 and fear of dying from COVID-19 were significantly positively correlated with anxiety and depression. Moreover, fear of contracting COVID-19 was positively and directly related to anxiety and depression which verifies the H1 and H3 hypotheses. In addition, fear of dying from COVID-19 was positively and directly related to anxiety supporting the H2 hypothesis, but was not directly related to depression. That fear of dying from COVID-19 was not determined to have a direct relationship with depression in this study was attributed to the relationship of depressive symptoms with the wish to die. The main source of anxiety, which was determined at a high rate during the pandemic, has been reported to be of becoming infected, and thus the focus was on the fear of spreading the infection to loved ones and them dying [9, 59]. Previous studies from Turkey have shown that the main reason for anxiety or stress among HCWs was the fear of contracting COVID-19 and spreading the virus to their families [60, 61]. With findings similar to those of the current study, in a meta-analysis of 91 studies from 36 countries, Alimoradi et al. [62] showed a moderate to strong association of fear of COVID-19 with stress, depression, and anxiety, and these relationships were seen more in HCWs than in the general population. In two studies from Turkey, one of HCWs [63] and the other of university students [64], a positive correlation was determined between COVID-19-related fear and anxiety and depression, and it was emphasized that fear of COVID-19 was a critically important precursor of mental health problems emerging in that period. It has been reported that there was a significant direct and indirect correlation between mental health and fear of COVID-19 in emergency nurses [65]. It is noteworthy that in an empirical study by Hauck et al. [66], there was seen to be evidence that higher COVID-related concerns led to increased fear levels via impaired fear learning and generalization, which could be a risk factor for the development of anxiety. As supported by the current study results, it has been shown that one of the leading reasons why COVID-19 is an important public health problem in terms of mental health and emotional status worldwide is the fear of being infected and dying caused by uncertainty about the virus in people regardless of their background [1, 25, 62, 67, 68].

### The mediating roles of traumatic stress and feeling lonely and distant from others

The results of the mediation analysis showed that fears of contracting and dying from COVID-19 affect anxiety and depression through traumatic stress and feeling lonely and distant from others. Hence, traumatic stress and feeling lonely and distant from others play parallel mediating roles between fear of COVID-19 and anxiety, and between fear of COVID-19 and depression.

First, traumatic stress was found to partially mediate the impacts of fear of contracting COVID-19 on anxiety and depression, which supported the H5 and H6 hypotheses. Traumatic stress also partially mediates the impact of fear of dying from COVID-19 on anxiety and depression, which verifies the H7 and H8 hypotheses. These indicate that the impact of disease fears on anxiety and depression are partly mediated by traumatic stress, and HCWs who are prone to stress are more likely to demonstrate the symptoms of anxiety and depression.

In several studies conducted in a similar period to that of the current study, COVID-related fear, stress, anxiety and depression were found to be at a high level and correlated with each other [69–72]. During the Severe Acute Respiratory Syndrome (SARS) epidemic, fear related to SARS was found to be positively correlated with post-traumatic stress symptoms among hospital staff [73]. A study from France reported that the baseline COVID-19 peritraumatic distress levels predicted the follow-up mental health conditions including post-traumatic stress, depression and anxiety, and explained roughly 14-20% of the variance [74]. Rodríguez-Hidalgo et al. [75] highlighted the complex relationship between fear of COVID-19, stress, anxiety and depression, and showed that fear, anxiety and stress were associated with depression. Moreover, it was determined that before most of the major depressive disorder (MDD) episodes, the individuals had experienced stressful life events [37]. Gómez Maquet et al. [76] reported that compared to control subjects, patients diagnosed with MDD had higher negative effects and perceived stress, higher frequency and poor perception of control of stressful life events, and negative appraisal of the situation. The formation of stress depends on cognitive and biological characteristics which affect how the individual interprets the situation and the outcomes [37]. Therefore, it has been emphasized that those with traumatic stress have difficulty regulating their fears [77]. Moreover, Wheatley [78] reported that as the “fight or flight” response created by fear and stress cannot be applied much under current living conditions, there can be adverse mental and psychological outcomes such as anxiety and depression.

Maeng and Milad [77], reported that traumatic stress, just like an exaggerated fear response, could alter the functioning of various neurobiological systems including the locus coeruleus/norepinephrine (NE) system and the corticotropin-releasing factor/hypothalamic-pituitary-adrenal (HPA) axis, often leading to increased responsiveness to future stressors. Abnormalities in the HPA-stress response, structural and functional damage which stress can create in neurons, the genetic and epigenetic effect, and reduced tolerance to stress by early life stressful events can lead to depression in particular, but it has been emphasized that the evidence is insufficient [79]. Although the underlying mechanisms have not yet been fully clarified, as shown in the current study, traumatic stress, whether acute or chronic, not only causes post-traumatic stress disorder but also anxiety and depression. The first question that comes to mind at this point is why people who experience similar adverse events do not develop psychopathology in all of these or at the same level. According to the diathesis-stress model, the reason for this is that the relationship between psychopathologies and stress is regulated by the predisposing characteristics of the individual, in other words, there is a greater probability of stress leading to psychological problems in those with a physiological, behavioural, or psychological predisposition [80, 81] This model was initially used for schizophrenia but was later shown to be applicable for other psychopathologies, including anxiety and depression [81, 82]. It has been argued that when social environment stressors with certain life stressors interact with pre-existing predisposing characteristics, this causes psychopathology by creating a vicious circle [83].

Moreover, feeling lonely and distant from others was found to partially mediate the impact of fear of contracting COVID-19 on anxiety and depression, which supported the H9 and H10 hypotheses. Feeling lonely and distant from others also partially mediated the impact of fear of dying from COVID-19 on anxiety and depression, which verifies the H11 and H12 hypotheses.

It has been reported that the feeling of loneliness is clearly different from the objective state of solitude, social isolation or being alone [84] and it is associated with increased psychiatric symptoms [85]. A systematic overview of forty systematic reviews published from 1950 to 2016, on the health consequences of loneliness and social isolation, reported consistent evidence linking them to worse mental health outcomes [44]. A substantial number of publications have also shown that during the COVID-19 pandemic, loneliness was positively associated with mental health [42, 86–90]. In a longitudinal study by Van der Velden et al. [45] conducted in Holland before and during the pandemic, it was reported that the feeling of loneliness increased in general together with COVID-19, and similar to the current study results, persistent loneliness was very strongly correlated with depression and anxiety symptoms. A recent study in the UK determined that those who reported often feeling lonely during the COVID-19 pandemic experienced 8-fold more mental health problems [91]. However, it has been observed that with support from friends and family, fear and loneliness decreased, and in parallel, depression and anxiety were alleviated [9]. Mahamid et al. [25] also reported that there was a negative relationship between mental health and social support during the COVID-19 pandemic, and fear of the pandemic played a mediating role in mental health problems.

In a study conducted in Turkey, a positive and significant relationship between the fear of death and disease transmission, uncertainty, loneliness and anxiety levels was observed in HCWs during the first wave of the pandemic [92]. Furthermore, a Norwegian study declared that loneliness was associated with both depression and anxiety even after controlling for all potential confounders and psychiatric diagnosis, and it was emphasized that the relationship to depression was more marked than the relationship to anxiety [43].

Loneliness is not specific to humans, and experimental studies of adult rodents have shown that isolation caused changes in prefrontal cortex myelinisation, in neurosteroid and growth factor concentrations, and functional alterations with an increase in HPA axis activity (glucocorticoid resistance, increased or blunted cortisol response etc.), and evidence has been presented that these changes were related to behaviours such as social withdrawal, aggressive behavior, anxiety and depressive behavior [93, 94].

Adam et al. [95] showed that in older adults who experienced feelings such as loneliness and sadness, feeling threatened and lack of control on the previous day, there was a correlation with a higher cortisol awakening response (CAR) the following day. This shows that the effect of loneliness on mental health is seen on the HPA axis. Some studies conducted during the COVID-19 pandemic support this relationship. For example, Haucke et al. [96], showed that during a lockdown period of COVID-19, momentary loneliness was associated with increased salivary cortisol indicating the activation of HPA axis which could lead to increased health risks such as anxiety and depression. In another short-term longitudinal study conducted in the first wave of the COVID-19 pandemic, it was reported that greater loneliness was associated with higher cortisol levels of waking and with a blunted cortisol awakening response (CAR) in young people even after controlling for potential covariates [97].

In loneliness, the regions of the brain related to attention and emotions are affected (insular cortex, prefrontal cortex, anterior cingulate cortex) and the activities of limbic regions which regulate motivation and the stress response, such as the amygdala and the hippocampus are altered, levels of plasma, salivary, and urinary cortisol are increased, and thus it has been reported that lonely individuals show a decreased response to positive social stimuli and an increased response to negative social stimuli, and exhibit increased alertness and attention [98]. This means that the effect of loneliness could be experienced more intensely in those with greater fear and at greater risk such as HCWs during the pandemic. In contrast, Nowland et al. [99] reported that lonely people did not exhibit a physiologically different response to certain social challenges, but showed higher sensitivity to social threat and perceived stress. These data demonstrate the need for further studies to more clearly show the predisposition to anxiety and depressive symptoms in lonely individuals.

### Implications

In this study, by establishing a mediator effect model, the effects of fear, stress, and loneliness were examined on the mental health of HCWs, who carried the main burden during the COVID-19 pandemic, and thus the subject was addressed in a broad context. To the best of our knowledge, this is the first study to have dealt with this subject in this multi-faceted way. Fear of the unknown in a pandemic is inevitable. Empirical evidence is presented of the effects on the development of anxiety and depression of the fear, stress, and loneliness experienced by HCWs because of their work. In addition, the after-effects of stress and loneliness were examined together with the antecedents in this study. The results highlight the need to pay attention to the great psychiatric burden of the pandemic. It has been shown in a sample of HCWs that psychosocial approaches to reduce stress and loneliness will improve mental health both directly and indirectly through mediating effects. For example, job security and social support that negatively contribute to the fear of HCWs can help to relieve the pressure on HCWs during such disease outbreaks. Although HCWs can experience more mental problems in these periods [62], the results could be applicable to the whole of society, and there is a need for further studies on this point. It can be understood that there is a need for the development of strategies by the government and hospital management to create a distance from the effects of pandemics that will lower psychological resistance and for these to be included in emergency action plans. This multi-directional approach will be of guidance in the development of multi-dimensional intervention strategies to increase the well-being of HCWs.

### Limitations

Despite the theoretical and practical contributions, there were also some limitations to this study. First, the study was cross-sectional in design and was conducted in the first wave of the COVID-19 pandemic. Therefore, causality could not be evaluated. It has been reported that the relationship of stress and loneliness with anxiety and depression could be bi-directional [37, 78, 79]. Anxiety and depression may also cause stress and loneliness. Therefore, detailed examination of the cause-and-effect relationship in the pandemic with longitudinal studies would shed more light on the subject. Another limitation was that as the results were based on self-reported statements there could have been recall bias, and because the questionnaire was completed online it was not possible to clarify the diagnosis especially for anxiety and depressive symptoms with a clinical interview. As the study was conducted only with secondary and tertiary-level HCWs in a single province, the results cannot be generalised for all HCWs, or nationally or globally. In addition, because a convenience sampling method was used, proper randomization was not achieved so the representation of the population could not be ensured. Moreover, fear, stress, and loneliness perceptions have cultural characteristics; for example, fear and stress can be more exaggerated in Asian culture [21]. The effects of the pandemic could have been felt differently because of the approach taken by different countries, the strategies applied, and the precautions implemented. For example in China and Italy, the effects of the pandemic were experienced very dramatically, and HCWs were especially negatively affected [100, 101]. A further limitation could be said to be that the previous mental health status of the participants was not taken into consideration. Due to the social restrictions under the COVID-19 conditions, a convenience sampling method was used rather than random sampling and the participants may have been those who wished to state their own ideas or to show that they had experienced more intense feelings. The mediator effect of stress and loneliness was examined in the relationship of fear of the disease with anxiety and depression, but it should not be forgotten that there could have been other mediators (job insecurity, job stress, burnout, coping strategies etc.) [65]. Thus, more comprehensive results could have been obtained with longitudinal studies and with examination of the predictors of anxiety and depression with models which considered the moderating effect of history of mental disease, gender, job satisfaction, and the unit in which the HCW was working.

## Conclusion

The COVID-19 pandemic caused extreme fear which exacerbated the global burden of mental health problems. This study presents a multi-dimensional perspective of the interactive mechanisms underlying the relationship between fear of being infected with COVID-19, fear of dying from COVID-19 and mental health in HCWs. The findings of this study can be considered to contribute to the literature on the subject of fear of disease and mental health, which are generally studied in the context of disease outbreaks, by examining the mediating effects of traumatic stress and loneliness among HCWs. The findings indicate that traumatic stress and loneliness played an important role in exacerbating the negative effects of fears of COVID-19 on anxiety and depression. The results provide insights for predicting HCWs who are more vulnerable to anxiety and depressive symptoms in order to implement the relevant prevention strategies for target groups and alleviate the detrimental impacts in a period of pandemic.

## Data Availability

The data of this study are available from the corresponding author upon reasonable request.

## Abbreviations

CAR: Cortisol Awakening Response
CI: Confidence Interval
COVID-19: Coronavirus Disease-2019
ETL: Evolutionary Theory of Loneliness
GUTS: Generalized Unsafety Theory of Stress
HADS: Hospital Anxiety Depression Scale
HCWs: Health Care Workers
HPA: Hypothalamic-Pituitary-Adrenal
ICC: Internal Consistency Coefficient
IES-R: Impact of Events Scale-Revised
ILO: International Labour Organization
MDD: Major Depressive Disorder
NE: Norepinephrine
NRS: Numeric Rating Scales
SARS: Severe Acute Respiratory Syndrome
SPSS: Statistical Package Program for Social Sciences
WHO: World Health Organization
